# Geographical disparities and determinants of infant mortality in Ethiopia: mapping and spatial analysis using EDHS data

**DOI:** 10.1186/s12887-023-04043-9

**Published:** 2023-05-05

**Authors:** Berhanu Awoke Kefale, Ashenafi Abate Woya, Abay Kassa Tekile, Getasew Mulat Bantie, Gizachew Yismaw Wubetu

**Affiliations:** 1 Department of Statistics, College of Natural and Computational Science, Jinka University, Jinka, Ethiopia; 2 Department of Statistics, College of Science, Bahir Dar University, Bahir Dar, Ethiopia; 3 Amhara National Regional State Public Health Institute, Bahir Dar, Ethiopia

**Keywords:** Infant mortality, Spatial analysis, Multilevel, Spatial effect, GLMM, Secondary data

## Abstract

**Background:**

Infant mortality remains a public health challenge in Ethiopia. Exploring infant mortality will aid in tracking the progress toward achieving sustainable development goals.

**Objective:**

The study aimed to explore the geographical variations and associated factors of infant mortality in Ethiopia.

**Method:**

A total of 11,023 infants from the 2016 Ethiopian Demographic and Health Survey (EDHS) data were extracted and included in the analysis. EDHS used a two-stage cluster sampling design with a census enumeration area as the primary sampling unit and households as the secondary sampling unit. Arc GIS software was used for spatial analysis using clusters for exploring geographical variations in infant mortality. A binary logistic regression was employed using R software to identify the significant determinants of infant mortality.

**Results:**

The study revealed that the spatial distribution of infant mortality was non-random in the country. Infants whose mothers not receiving ANC (AOR = 1.45; 95%CI: 1.17, 1.79), not breastfed status (AOR = 3.94; 95%CI: 3.19, 4.81), poor wealth index (AOR = 1.36; 95%CI: 1.04, 1.77), male infants (AOR = 1.59; 95%CI: 1.29, 1.95), birth order of six or above (AOR = 3.11; 95%CI: 2.08, 4.62), small birth size (AOR = 1.27; 95%CI: 1.26, 1.60), birth spacing [(≤ 24 months (AOR = 2.29; 95%CI: 1.79, 2.92), 25–36 months (AOR = 1.16; 95%CI: 1.12, 1.49)], multiple births (AOR = 6.82; 95%CI: 4.76, 10.81), rural residence (AOR = 1.63; 95%CI: 1.05, 2.77) and regions [Afar (AOR = 1.54; 95%CI: 1.01, 2.36), Harari (AOR = 1.56; 95%CI: 1.04, 2.56), and Somali (AOR = 1.52; 95%CI: 1.03, 2.39)] were the determinants of infant death in Ethiopia.

**Conclusions:**

There is a great geographical disparity in infant mortality rates across regions. Afar, Harari, and Somali regions were verified as hot spot areas. ANC usage, breastfed status, wealth index, sex of the infant, birth order, birth size, birth spacing, birth type, residence, and region were the determinants of infant death in Ethiopia. Therefore, appropriate interventions need to be implemented in the hot spots to alleviate the risk factors for infant mortality.

## Introduction

Infant mortality is the most accurate national measure of health, socioeconomic progress, and standard of living [1–4]. Despite several interventions to prevent infant mortality, it remains a leading public health concern worldwide, mainly in the developing world [5–8]. Ethiopia is one of the countries with a high infant mortality rate (48 per 1,000 live births in 2016), which is attributed to 75% of all under-5 deaths [9, 10]. Though nearly 80% of infant deaths are from preventable causes, it is still a challenging public health problem to attain sustainable development goals [9, 11, 12].

Recent studies revealed that lack of proper child care, ANC follow-up, low birth weight, low breastfeeding practice, maternal complications, hand washing with soap, the mother’s perception of modern medical treatments, birth order, and birth interval were the individual-level determinants of infant mortality [12–19]. Other studies also showed that the mother’s age and education, the childbirth order, piped water and sewers, geographical access to health care, and the sex of the infant were predictors of infant mortality [20–22]. Infant mortality might vary at the community level due to variations in access to the nearest health and educational institutions, means of communication, poverty rate, access to skilled maternal health services, and presence of community media [13, 14, 22–27].

Exploring geographic variations and inequalities in infant mortality will help to track progress towards the Sustainable Development Goals (SDGs). This information will help policymakers and planners give emphasis and strategically allocate scarce resources to achieve the SDGs. Hence, this study aimed to explore the geographical variations of infant mortality and the determinants in regions of the country and identify localities with high infant mortality by spatial analysis using the 2016 Ethiopian Demographic and Health Survey data.

## Methods

### Study design, area, and period

This cross-sectional study was done using the 2016 EDHS dataset. The data was accessed from the CSA after securing permission through an online request explaining the objective of our study. The survey covered all nine regions and two city administrations of Ethiopia, and regions are divided into 68 zones, 817 districts, and 16,253 kebeles [28].

### Source of data

The data used in this article was gotten from the Ethiopia Demography and Health Survey (EDHS) 2016, which was accessed at the MEASURE DHS website after securing a formal request to the MEASURE DHS program.

### Data collection tools and procedures

The sample was selected using stratified, two-stage cluster sampling with enumeration areas (EAs) as the sampling units for the first stage. The sample included 643 EAs. Households were contained in the second stage of sampling. A complete listing of households was carried out in each of the 643 selected EAs, and all households in all selected EAs were interviewed in the survey. The methodology for sample selection and data collection is explained in the EDHS 2016 report [29]. A cluster in DHS surveys is the smallest geographical area comprised of a number of adjacent households. A cluster may correspond to an EA or a segment of a large EA with well-defined boundaries. However, an EA is a geographic area covering, on average, 181 households.

### Data measurement

The outcome variable for the study was infant mortality. The characteristics at the individual level **(**mother’s age, education, occupation, marital status, sex of household head, wealth index, family size, breastfed status, ANC usage, place of delivery, birth size, mode of delivery, type of birth, sex of child, birth interval, and birth order) and community level characteristics (residence and region) were the explanatory variables. From the total of 643 EAs, 622 were included. A total of 11,023 live births within the five years preceding the 2016 EDHS were included.

### Statistical data analysis

Data analysis was done using R and Arc GIS software. The infant mortality rate for each of the selected EAs and regions (with a 95% confidence interval) was calculated by taking the complex survey sampling design into account.

### Geospatial data processing

The infant mortality dataset was arranged in spatial form using a hierarchical logistic regression model. A spatial model was used to discern spatial patterns of infant mortality and to explore different spatial covariance structures. A spatial autocorrelation (Global Moran’s I) statistic was used to measure the overweight patterns in the study area. A statistically significant Moran’s I (p < 0.05) was used as an indicator of spatial autocorrelation. Moreover, a spatial interpolation technique was used to predict the overweight of the unsampled areas in the country based on sampled EAs using ordinary Kriging spatial interpolation methods.

## Results

### Infant mortality rate disparities

**Fig. 1:**
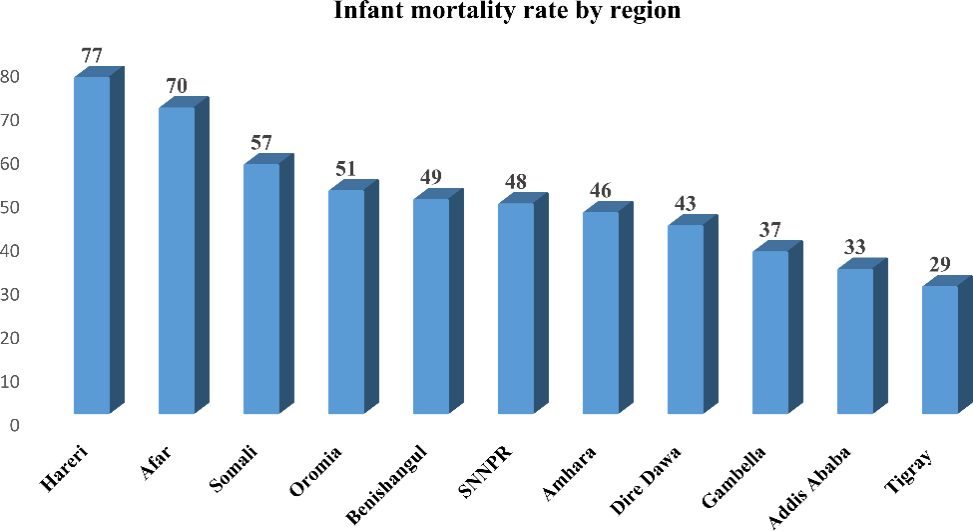
Regional infant mortality rate disparities in Ethiopia, 2016

In Ethiopia, the infant mortality rate in 2016 was 48 per 1,000 live births, with significant differences between regions within the country. Harari region had the highest rate of infant mortality (76.9 per 1,000 live births), while Tigray region had the lowest rate of infant mortality (29.3 per 1,000 live births) (Fig. 1).

### Spatial analysis of infant mortality

**Fig. 2:**
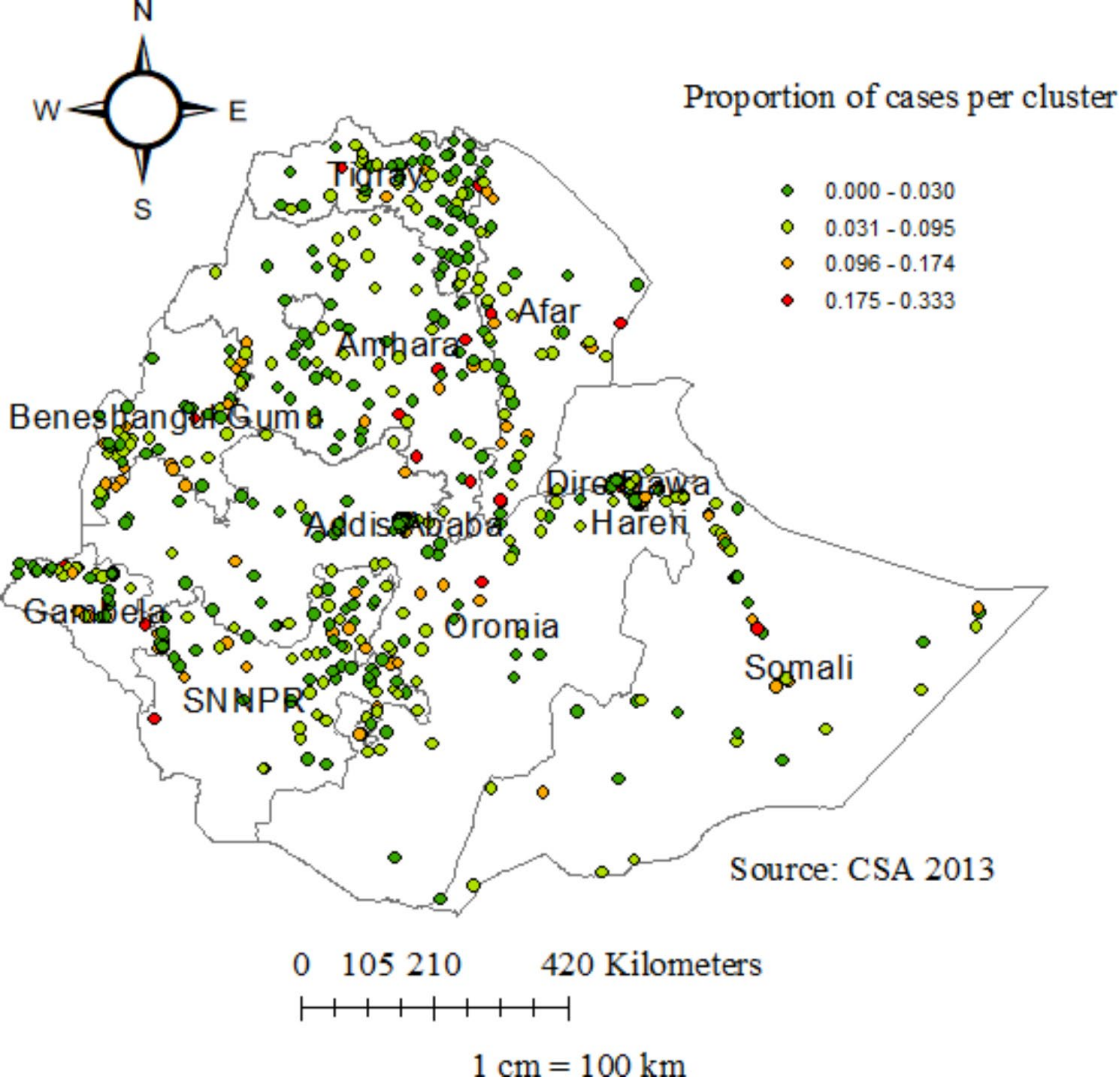
Spatial distribution of infant mortality among infants in Ethiopia, 2016

The spatial kriging interpolation analysis was used to predict infant mortality for unsampled areas of the country based on sampled enumeration area measurements. This study revealed that the distribution of infant mortality was significantly clustered in Ethiopia (Global Moran’s Index = 0.1546; p-value = 0.0185). Each point on the map characterizes one enumeration area, with the proportion of infant mortality in each cluster. The green color indicates the areas with a low proportion of infant mortality, whereas the red color indicates the enumeration areas with a high proportion of infant mortality. The highest proportions of infant mortality occurred in a majority part of the Harari region, the east part of Afar, the border of Benishangul Gumuz and Oromia, the central part of Somali, the border of Amhara and Benishangul Gumuz, and the central part of Oromia. Whereas the low proportion of infant mortality was accumulated in Tigray, Dire Dawa, Gambela, the entire part of Addis Ababa, and the northeast part of the South Nation Nationalities and Peoples region (SNNPR). The circles are the centroids, which are the spatial mean and the average location of all points and can also be thought of as the balance point of a set of points (Fig. 2).

**Fig. 3:**
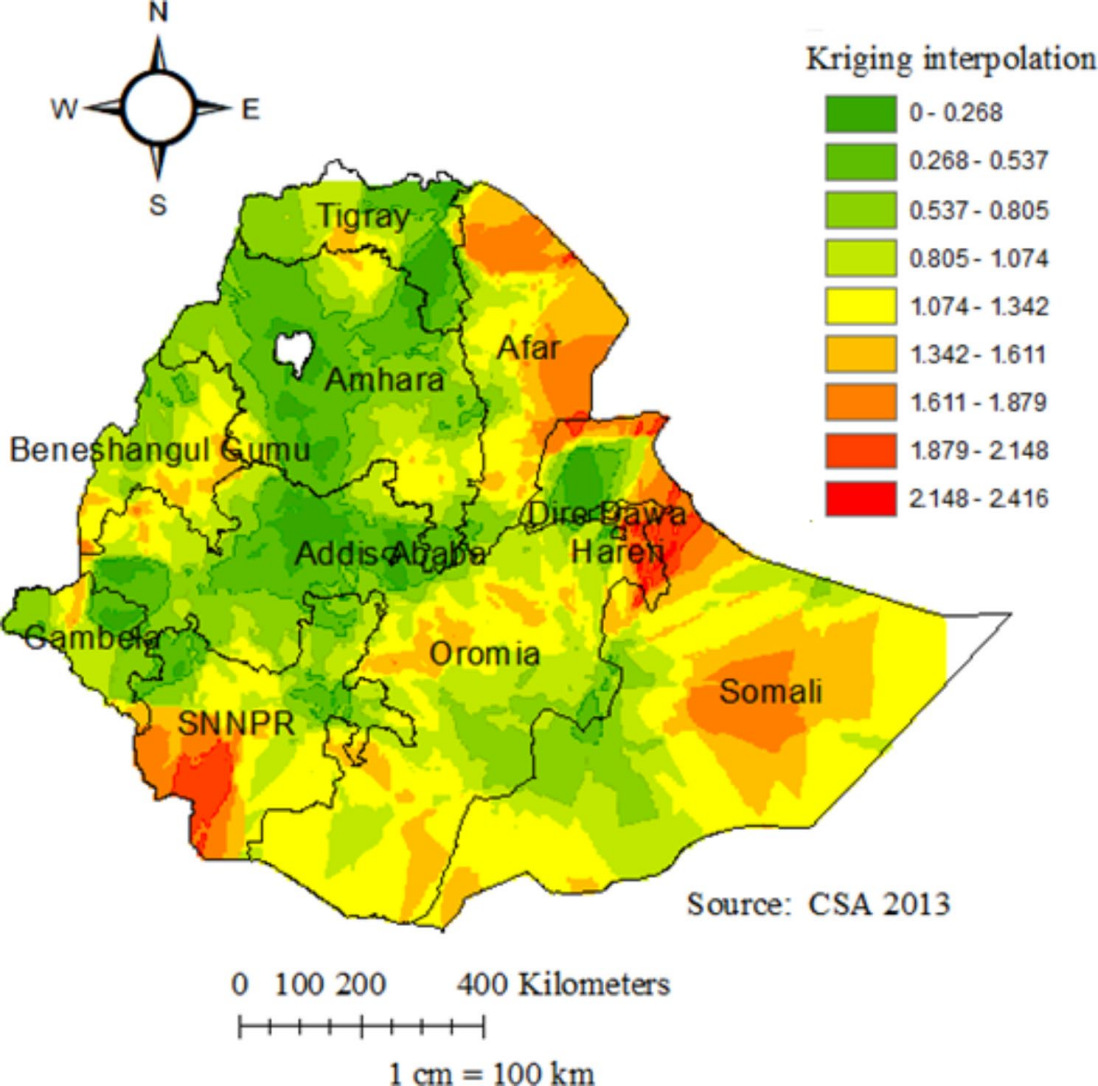
Kriging interpolation of infant mortality among infants in Ethiopia, 2016

The spatial kriging interpolation analysis was used to predict infant mortality in unsampled areas of the country based on sampled enumeration area measurements. The red color represented the prediction of high-risk areas. Afar, Somali, Harari, the Southwest SNNPR, East Benishangul Gumuz, and the south and central parts of Oromia were predicted as high-risk areas compared to other regions. Whereas Tigray, Amhara, Addis Ababa, Northwest Oromia, North Somali, Dire Dawa, Northwest Benishangul Gumuz, Northeast SNNPR, border of Gambela and SNNPR, the border of Gambela, and Oromia were predicted as having less risk for infant mortality (Fig. 3).

### Determinants of infant mortality

In the model, ANC usage, breastfed status, wealth index, sex of the infant, birth order, birth size, birth spacing, birth type, residence, and region were the determinants of infant death in Ethiopia.

Infants of mothers who did not receive ANC during the last pregnancy had about 1.5 (AOR = 1.45; 95% CI: 1.17, 1.79) times higher odds of death in their first year of life compared with infants whose mothers did receive ANC services. Similarly, for infants, who were not breastfed, the odds of death were nearly fourfold (AOR = 3.94; 95%CI: 3.19, 4.81) higher compared to infants who were breastfed.

For mothers who belong to the poor wealth index, the odds of their infant’s death were nearly 1.4 (AOR = 1.36; 95% CI: 1.04, 1.77) times higher compared to mothers who belong to the rich wealth index. Likewise, for mothers whose infants were male, the odds of their infant death were (AOR = 1.59; 95% CI: 1.29, 1.95) times higher compared to mothers having female infants.

Infants with birth orders of six and above had approximately 3.11 times the odds of death as compared to the reference group (AOR = 3.11; 95%CI: 2.08, 4.62). Similarly, Infants with birth orders of six and above). Similarly, the odds of infant death among infants with small birth sizes were 1.27 (AOR = 1.27; 95% CI: 1.26, 1.60) times higher compared to large-birth-sized infants.

The estimated odds of death among infants born with a preceding birth spacing of less than or equal to 24 months were 2.29 (AOR = 2.29; 95% CI: 1.79, 2.92) times more likely to die as compared to infants born with a birth spacing of more than 36 months. Whereas the estimated odds of dying infants born with a preceding birth spacing of between 25 and 36 months were 1.16 (AOR = 1.16; 95% CI: 1.12, 1.49) times more likely to die as compared to infants born with a birth spacing of more than 36 months in the same clusters, keeping other covariates constant. Moreover, the odds of infant mortality among multiple births were 6.82 (AOR = 6.82; 95% CI: 4.76, 10.81) times higher compared to singletons.

The odds of infant mortality among rural residents were 1.63 (AOR = 1.63; 95% CI: 1.05, 2.77) times higher compared to urban residents. Likewise, infants living in Harari, Afar, and Somali regions had a higher chance of mortality [Afar (AOR = 1.54; 95% CI: 1.01, 2.36), Harari (AOR = 1.56; 95% CI: 1.04, 2.56), and Somali (AOR = 1.52; 95% CI: 1.03, 2.39)] compared with infants living in Tigray region. Furthermore, there are spatial autocorrelation of infant mortality between clusters (P = 0.0028) and there was a spatial variable correlation with infant mortality (r = -0.5778) indicating that clusters with a low incidence of infant mortality were surrounded by clusters with a high incidence of infant mortality **(**Table 1**).**

**Table 1:** Parameter estimate for models in the study using individual-level, community-level, and spatial autocovariance variables

Variables	Category	*β* Estimate (95%CI)	SE	AOR (95%CI)
**Intercept**		-4.9958* (-5.89, -4.09)	0.46	0.007 (0.003, 0.017)
**ANC usage**	**No**	0.3739** (0.16, 0.58)	**0.13**	**1.45 (1.17, 1.79)**
	Yes	0		1.00
**Breastfed status**	**No**	1.3701** (1.16, 1.57)	**0.14**	**3.94 (3.19, 4.81)**
	Yes	0		1.00
**Wealth index**	Medium	-0.2736 (-0.58, 0.03)	0.16	0.76 (0.56, 1.03)
	**Poor**	0.3074* (0.04, 0.57)	**0.14**	**1.36 (1.04, 1.77)**
	Rich	0		1.00
**Sex of child**	**Male**	0.4668** (0.26, 0.67)	**0.13**	**1.59 (1.29, 1.95)**
	Female	0		1.00
**Birth order**	**6 and more**	1.1331** (0.73, 1.53)	**0.22**	**3.11 (2.08, 4.62)**
	4–5	0.1880 (-0.20, 0.57)	0.22	1.21 (0.82, 1.77)
	2–3	0.3262 (-0.01, 0.64)	0.17	1.39 (0.99, 1.89)
	First	0		1.00
**Birth size**	Average	-0.2798 (-0.51, 0.04)	0.12	0.76 (0.6, 1.04)
	**Small**	0.2417** (0.23, 0.47)	**0.13**	**1.27 (1.26, 1.60)**
	Large	0		1.00
**Birth spacing (in months)**	**≤** **24**	0.8324** (0.58, 1.07)	**0.15**	**2.29 (1.79, 2.92)**
	**25–36**	0.1497** (0.11, 0.40)	**0.13**	**1.16 (1.12, 1.49)**
	> 36	0		1.00
**Type of birth**	**Multiple**	1.9205** (1.56, 2.38)	**0.21**	**6.82 (4.76, 10.81)**
	Single	0		1.00
**Sex of household head**	Male	0.7174 (-0.37, 1.07)	0.18	2.05 (0.69, 2.92)
	Female	0		1.00
**Residence**	**Rural**	0.4867** (0.05, 1.02)	**0.29**	**1.63 (1.05, 2.77)**
	Urban	0		1.00
**Region**	Addis Abeba	0.2438 (-0.24, 0.73)	0.25	1.28 (0.79, 2.08)
	**Afar**	0.4322* ( 0.01, 0.86)	**0.22**	**1.54 (1.01, 2.36)**
	Amhara	0.4276 (-0.20, 1.06)	0.32	1.53 (0.82, 2.89)
	Benishangul Gumez	0.7658 (-0.29, 1.24)	0.24	2.15 (0.75, 3.46)
	Dire Dawa	0.6528 (-0.06, 1.24)	0.30	1.92 (0.94, 3.46)
	Gambela	0.3823 (-0.17, 0.93)	0.28	1.47 (0.85, 2.54)
	**Harari**	0.4454* (0.04, 0.94)	**0.25**	**1.56 (1.04, 2.56)**
	Oromia	0.4293 (-0.18, 1.04)	0.31	1.54 (0.84, 2.83)
	SNNPR	0.3189 (-0.31, 0.94)	0.32	1.37 (0.73, 2.56)
	**Somali**	0.4160* (0.03, 0.87)	**0.23**	**1.52 (1.03, 2.39)**
	Tigray	0		1.00
**Si** (P-value = 0.0028)		-0.5778*(-0.95, -0.20)	0.19	

## Discussion

The Ethiopian government has been strongly striving to improve child health for the last two decades [30]. But infant mortality remains a significant public health issue in the country [31]. In Ethiopia, the infant mortality rate was 48 per 1,000 live births in 2016, with significant differences between regions within the country.

The current spatial analysis using different methods consistently verifies hot and cold spot areas of infant mortality among infants in Ethiopia. Moreover, the study identified the determinants of infant mortality in Ethiopia.

The spatial analysis showed that the Afar, Harari, and Somali regions of Ethiopia were statistically significant hotspot areas for infant mortality. A possible justification could be variations in ANC utilization across regions. The lowest ANC utilization rate was reported in the hot spot areas (Afar and Somali regions) as compared to the cold spot areas [28]. This could be attributed to the discrepancy in the distribution of maternal health services and environmental factors across the area [32]; This implies that identifying regions with high infant mortality is important for prioritizing areas for analysis of cause and planning of remedial actions. In contrast, the cold spot areas were observed in the entire part of Addis Ababa, the central part of the Oromia and Amhara regions. The possible justification could be that these regions are urban as compared to the hotspot areas. They have good access to health facilities, and mothers may be aware of ANC utilization and its benefits compared to other regions.

Infant mortality is significantly associated with ANC use. Infants born to mothers who did not use ANC during pregnancy had a higher risk of infant death than those born to mothers who used ANC. This finding is consistent with studies conducted in Ethiopia [33], Pakistan [34] and Brazil [35]. This could be related to a lack of maternal awareness of neonatal and infantile danger signs. The other possible justification for the finding might be linked with bad cultural taboos, as the mothers could delay seeking health care and end up with poor health outcomes.

The odds of dying among infants with a sixth or higher order of birth were more likely than those of first-born infants. This finding is consistent with the studies conducted in Ethiopia [33] and South Africa [36]. Similarly, the risk of death among male infants was higher than that of female infants. This result is in line with studies conducted in Ethiopia [37] and Bangladesh [38].

The odds of death among infants born with a preceding birth spacing of between 25 and 36 months and less than or equal to 24 months were higher as compared to infants born with a birth spacing of more than 36 months. This result is similar to studies conducted in Ethiopia [39] and Nepal [40].

This study also demonstrated that the odds of death among multiple births were higher than those among singletons. This finding is consistent with studies conducted in Ethiopia [37, 41], Kenya [42], and Brazil [43]. The possible justification for this could be that multiple births are at high risk for numerous negative birth outcomes, and these outcomes contribute to a higher rate of mortality during the infancy period [44].

This study indicated that breastfeeding status has a significant association with infant mortality. Among mothers who did not breastfeed their child, the odds of infant death were higher as compared with mothers who did. This finding is supported by previous studies done in Ethiopia [22] and Kenya [45].

The wealth index also has a significant association with infant mortality. Among mothers who belong to the poor wealth index, the odds of infant death were higher as compared to their counterparts. This result is in line with studies done in Bangladesh [38].

The odds of infant death among rural infants were higher as compared to their counterparts among urban residents. This finding is consistent with studies conducted in Ethiopia [37], Nigeria [46], and Pakistan [34]. The reason may be that mothers living in rural areas lack access to health institutions to have ANC follow-ups or lack media exposure, which in turn affects their knowledge and practice of care for the infant.

The odds of dying during the infancy period in the Afar, Somali, and Harari regions were higher as compared to infants from the Tigray region. This finding is in line with a study conducted in Ethiopia [41]. The difference in mortality between regions may be due to variations in service accessibility and coverage. The national Universal Health Coverage (UHC) service capacity and access coverage were lowest in the Somali and Afar regions as compared to the Tigray region [47]. These regions are also known for socioeconomic vulnerability and food insecurity, which lead to malnutrition and infant deaths [48].

This study reveals that the spatial variable has a negative significant effect where clusters with a low incidence of infant mortality were usually surrounded by clusters with a high incidence of infant mortality. Whereas, clusters with a high incidence of infant mortality were usually surrounded by clusters with a low incidence of infant mortality, which is in line with studies in Brazil [49].

Ethiopia is one of the countries with high infant mortality and great disparities between regions. The high infant mortality rate was observed in the three regions (Harari, Afar, and Somali) of Ethiopia. This implicated that the high infant mortality rate is highly correlated with poor child care practices at the household and community level. It could also be connected with a weak health care delivery system and poor health infrastructure in the country. Hence, the policy designed to protect infants from dying should be revised to attain sustainable developmental goals.

### Limitations of the study

As the current study was based on secondary data analysis, there is the possibility of reporting and recall bias for retrospective data relying on memory of a past event. Health service use- related factors were collected only for the most recent births, limiting the scope of analysis. Moreover, there were omissions of values for some variables, limiting the exhaustive consideration of the determinants.

## Conclusions

With the combined efforts of the country’s policies and programs to improve child health, the infant mortality rate dropped significantly in Ethiopia. However, great geographical disparities in infant mortality rates still persist across regions. Harari, Afar, and Somali regions were verified as hot spot areas. ANC usage, breastfed status, wealth index, sex of the infant, birth order, birth size, birth spacing, birth type, residence, and region were the determinants of infant death in Ethiopia. Therefore, reducing infant mortality via appropriate strategies for the attainment of the sustainable development goal should be a priority concern.

## Data Availability

The dataset used for analysis is available in the EDHS Program repository at the following link: http://dhsprogram.com. Data will be available upon request from the corresponding author.

## Abbreviations

ANC: Antenatal Care, :
Arc GIS: Aeronautical Reconnaissance Coverage Geographic Information System
CI: Confidence Interval
CSA: Central Statistical Agency
EAs: Enumeration Areas
EDHS: Ethiopian Demographic Health Survey
GLMM: Generalized Linear Mixed Model
GPS: Global Positioning System.
